# Antireflux Ureteral Stents Prevent Stent-Related Symptoms: A Meta-Analysis of Randomized Controlled Trials

**DOI:** 10.7759/cureus.49375

**Published:** 2023-11-24

**Authors:** Mohammed Zain Ulabedin Adhoni, Ammar Al Homsi, Zubeir Ali, Ahmad Almushatat

**Affiliations:** 1 Urology, Royal London Hospital, London, GBR; 2 Urology, Surgical Subspecialties Institute, Cleveland Clinic Abu Dhabi, Abu Dhabi, ARE

**Keywords:** jj stent, stent-related-pain, stent-related-symptoms, antireflux stent, ureteral stent

## Abstract

Ureteral stents are widely used in urological care, but they are often associated with adverse stent-related symptoms (SRS), such as painful urination, elevated urinary frequency, and abdominal discomfort. Antireflux ureteral stents have been developed to reduce stent-related pain and reflux by minimizing vesicoureteral reflux (VUR).

This systematic review and meta-analysis of randomized controlled trials (RCTs) was undertaken to assess the efficacy of antireflux ureteral stents in mitigating SRS compared to conventional urethral stents. Our study included a total of 269 cases from three RCTs.

The meta-analysis showed that antireflux ureteral stents were significantly more effective than standard stents in reducing SRS, including stent-related pain (odds ratio (OR): 4.80, 95% CI: 2.77, 8.31, p <0.00001), severe stent-related pain (OR: 8.35, 95% CI: 2.12, 32.89, p=0.002), flank pain while urinating (OR: 5.98, 95% CI: 3.35, 10.68, p <0.00001), and severe flank pain while urinating (OR: 15.79, 95% CI: 2.91, 85.57, p=0.001). There was no significant difference in the rates of postoperative creatinine abnormality or postoperative hydronephrosis between the two groups.

Therefore, antireflux ureteral stents are more effective than standard stents in reducing SRS. This suggests that antireflux ureteral stents should be considered for patients undergoing ureteral stenting.

## Introduction and background

In urological care, ureteral stents are widely used for various clinical applications, such as alleviating ureteric obstructions, aiding post-surgical drainage, and serving as a supplementary tool in endourological procedures [1]. However, they are often linked with a series of adverse stent-related symptoms (SRS), including but not limited to painful urination, elevated urinary frequency, and abdominal discomfort [2,3]. Several studies in the literature estimated a range of incidences for various symptoms: irritative voiding symptoms such as frequency (50%-60%), urgency (57%-60%), and dysuria (40%) are noted, along with a feeling of incomplete emptying (76%). Pain is often localized to the flank (19%-32%) or the suprapubic area (30%). Incontinence is also reported, and hematuria is seen in 25% of instances [2-4]. These adverse effects can significantly compromise the patient's quality of life and may sometimes necessitate premature stent extraction or additional medical interventions [3]. A retrospective study by Geavlete et al. noted that irritative bladder symptoms occurred in 32.7% of the 50,000 cases evaluated. Of these, 4.7% of overall cases warranted intervention with specific medications, and in 0.074% of total cases, stent removal was necessary [4].

There have been advances in ureteral stent design, which include antireflux membrane valves, polymeric flap valves, and many others. These stents aim to reduce SRS by minimizing vesicoureteral reflux, which is commonly observed with conventional stents [1]. The antireflux membrane valve, highlighted by Ecke et al. [5, 6], is clinically accessible and employs a silicone membrane situated at the vesical end of the double J stent (JJ stent/DJS), forming a one-way valve, thereby obstructing intraluminal vesicoureteral reflux (VUR) upon escalation of bladder pressure. Introduced by Park et al. [5, 7], the polymeric flap valve is affixed to the vesical edge of the DJS, leveraging the differential pressures between the bladder and ureter to prevent retrograde urine flow; its efficacy was substantiated in vivo by Kim et al. [5, 8]. An innovative design demonstrated by Vogt et al. avoids the traditional bladder pigtail of the DJS in favor of a silicone piece with antireflux functionality positioned at the ureteral orifice [9,10]. The BraidStent®, developed by Soria et al., emerges as an intraureteral stent aiming to maintain the integrity of the VUR to prevent intraluminal and extraluminal reflux [11,12]. These designs represent diverse strategies and materials to address the challenges posed by VUR, each presenting varying levels of success and prospective utility in clinical environments.

While prior studies, such as those conducted by Koprowski et al. and Miyaoka et al., have investigated the efficacy of antireflux stents (AR JJs), the results are marked by considerable variability [2,3]. Hence, a clear consensus has not yet been reached on the effectiveness of antireflux ureteral stents in the prevention of SRS [1,2].

The primary objective of this meta-analysis is to comprehensively review existing literature comparing the efficacy of antireflux ureteral stents with standard stents in the mitigation of SRS. By analyzing data from multiple randomized controlled trials (RCTs), this meta-analysis offers a comprehensive assessment that could significantly influence clinical practice and guide future research efforts in this critical field.

## Review

Methods

This study was registered in the International Prospective Register of Systematic Reviews (PROSPERO) with the identification number CRD42023468281. Authors M.A. and A.A.H. conducted a bibliographic search of Medical Literature Analysis and Retrieval System Online (MEDLINE) and Google Scholar on September 30, 2023, with the search terms ("antireflu*" OR "valve" OR "reflux") AND ("JJ stent*" OR "Ureteric Stent*" OR "Ureteral Stent*" OR "DJ Stent*") on MEDLINE with the restriction to include only randomized controlled trials. On Google Scholar, our search terms were ("antireflux" OR "anti-reflux") AND ("JJ stent" OR "Ureteric Stent" OR "Ureteral Stent" OR "DJ Stent") AND ("randomized controlled trial" OR "controlled trial" OR "RCT").

**Figure 1:**
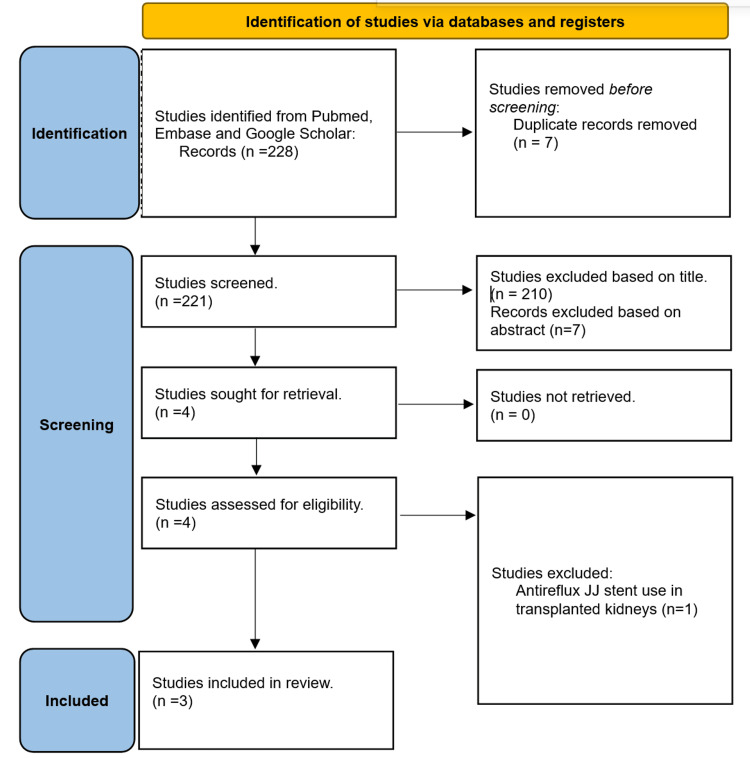
PRISMA flowchart of systematized search with included studies PRISMA: Preferred Reporting Items for Systematic Reviews and Meta-Analyses

Our population of interest was patients undergoing ureteral stent placement, with a comparison of the outcomes of standard JJ (sJJ) stent insertions versus AR JJ stent insertions. We only included RCTs. The exclusion criteria included studies with a focus on antireflux stents in transplanted kidneys, studies published in languages other than English, and conference abstracts. A Preferred Reporting Items for Systematic Reviews and Meta-Analyses (PRISMA) flowchart summarizes the selection process (Figure 1).

**Figure 2:**
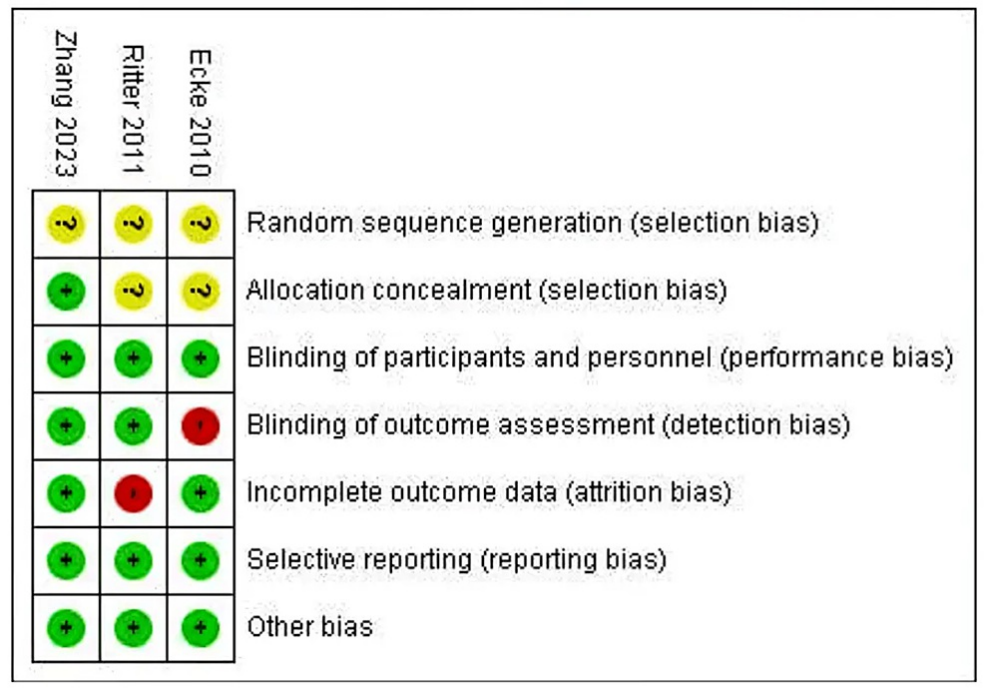
Risk of bias summary [6,13,14]

**Figure 3:**
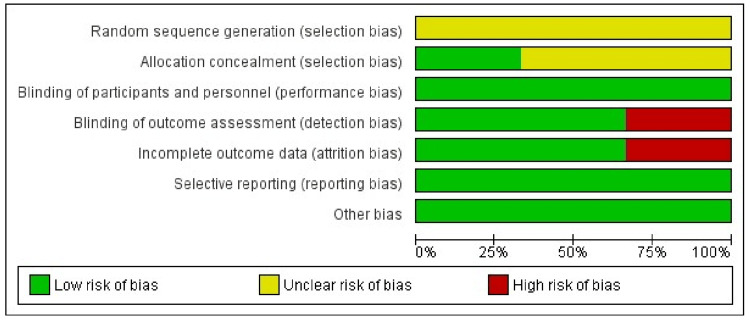
Risk of bias graph

Any discrepancies in the article selection and exclusion between M.A. and A.A.H. were resolved through discussion. The authors, M.A. and A.A.H., independently further conducted a stratified risk of bias (RoB) assessment, as summarized in Figures 2-3.

A total of three RCTs are included in this review.

Data analysis was performed in Review Manager V5.4 (RevMan) (Computer Program), The Cochrane Collaboration, 2020). Higgins’ I2% test was employed to test heterogeneity, using 50% as a cutoff value. A fixed-effects model was used for homogenous data and a random-effects model for heterogeneous data. Dichotomous data such as stent-related pain were reported using odds ratios (OR) with a 95% CI. The resulting values with associated p-values <0.05 were considered significant.

Results

A total of three randomized controlled trials are included in this review, which included a total of 269 cases, of which 130 underwent sJJ stent insertion and 139 underwent AR JJ stent insertion. The study characteristics are summarized in Table 1 [6,13,14].

**Table 1:** Study characteristics of the included randomized controlled trials [6,13,14] sJJ: standard JJ; AR JJ: antireflux JJ; VUR: vesicoureteral reflux

Author (year)	Total population (sJJ vs AR JJ)	Type of standard stent	Type of antireflux stent	Inclusion criteria	Exclusion criteria
Zhang et al. (2023) [13]	107 (56 vs. 51)	5F 26 cm standard ureteral stent (KYB Medical, Shenzhen, Guangdong, China)	5F 26 cm antireflux ureteral stent (KYB Medical, Shenzhen, Guangdong, China)	Adult patients (>18 years old) with ureteral stones (5–20 mm measured on CT) needing surgical intervention and ureteral stent insertion	(1) Congenital VUR or other reasons causing VUR before; (2) Chronic flank pain and suprapubic pain before; (3) Low quality-of-life state caused by other pre-existing diseases; (4) Bilateral stent insertion; (5) Pre-stented cases; (6) Concomitant medications known to influence stent-related symptoms, including alpha-blockers, antimuscarinics, and beta 3-agonist; and (7) Ureteral stricture or ureteral trauma.
Ritter et al. (2011) [14]	29 (13 vs 16)	Conventional JJ stent (7 Fr., 26 cm, ST-197726, polyurethane, Urovision®, Bad Aibling, Germany)	Antirefluxive JJ stent (7 Fr., 26 cm, UF-110726 polyurethane, Urovision®, Bad Aibling, Germany)	Patients with acute hydronephrosis due to ureteral calculi requiring internal drainage	(1) Urinary tract infection; (2) Known VUR; (3) Patients under 18 years of age; (4) Patients with a history of ureteral manipulation, including stent placement or ureterorenoscopy
Ecke et al. (2010) [6]	133 (61 vs 72)	JJ ureteral stent sets made of polyurethanes, 28-cm (Teleflex Medical, GmbH, Kernen, Germany)	JJ ureteral stent with antireflux valve, made of polyurethanes, 28-cm (Teleflex Medical, GmbH, Kernen, Germany)	All patients referred for treatment of hydronephrosis	Nil

Stent-related pain

**Figure 4:**
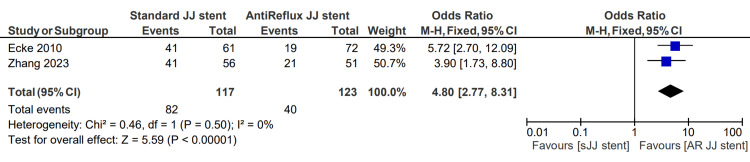
Stent-related pain [6,7] sJJ: standard JJ; AR JJ: antireflux JJ

Stent-related pain was described in two studies, totaling 117 patients in the sJJ stent group and 123 in the AR JJ stent group. Stent-related pain rate for patients having sJJ stents is 70.0%, compared with 32.5% for those with AR JJ stents. The associated OR for stent-related pain was 4.80 (2.77, 8.31), p <0.00001. This finding is displayed in Figure 4 [6,13].

Severe stent-related pain

**Figure 5:**
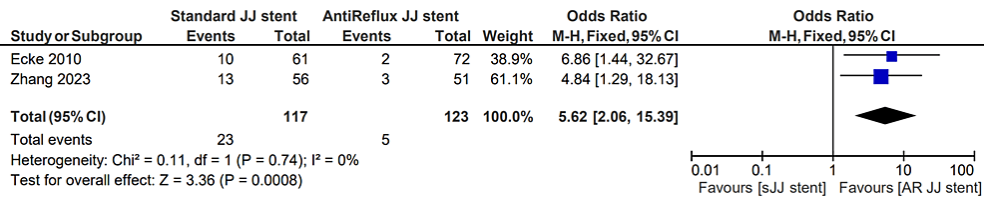
Severe stent-related pain [6,13] sJJ: standard JJ; AR JJ: antireflux JJ

Patients complaining of stent-related pain “often” or “very often” were classified as having severe symptoms. The severity of stent-related pain was described in two studies, totaling 117 patients in the sJJ stent group and 123 in the AR JJ stent group. The severe stent-related pain rate for patients having sJJ stents is 13.7%, compared with 1.6% for those with AR JJ stents. The associated OR for severe stent-related pain was 5.62 (95% CI: 2.06, 15.39, p=0.0008). This finding is displayed in Figure 5 [6, 13].

Flank pain while urinating (reflux symptoms)

**Figure 6:**
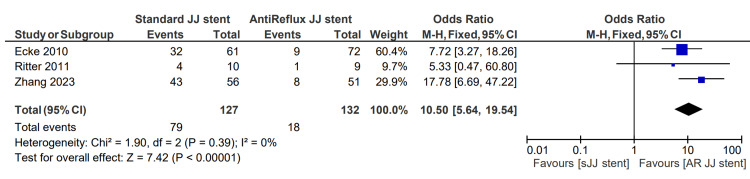
Flank pain during urination (reflux symptoms) [6,13,14] sJJ: standard JJ; AR JJ: antireflux JJ

Flank pain while urinating was described in three studies, totaling 127 patients in the sJJ stent group and 132 in the AR JJ stent group. The reflux symptoms rate for patients having a sJJ stent is 62.2%, compared with 13.6% for those with an AR JJ stent. The associated OR for reflux symptoms was 10.50 (5.64, 19.54), p <0.00001 [6,13,14]. This finding is displayed in Figure 6.

Severe reflux symptoms

**Figure 7:**
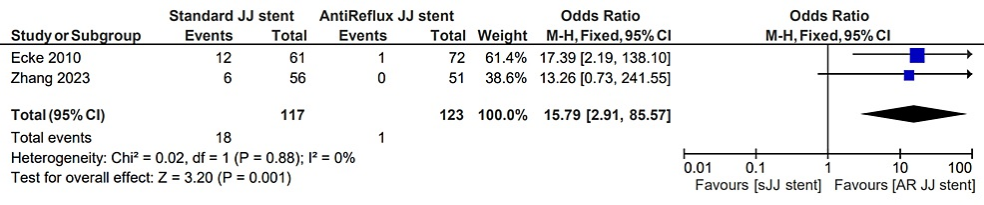
Severe reflux symptoms [6,13] sJJ: standard JJ; AR JJ: antireflux JJ

Patients complaining of reflux symptoms “often” or “very often” were classified as having severe symptoms. The severity of reflux symptoms was described in two studies, totaling 117 patients in the sJJ stent group and 123 in the AR JJ stent group. The severe reflux symptoms rate for patients with sJJ stents is 15.4%, compared with 0.8% for those with AR JJ stents. The associated OR for severe reflux symptoms was 15.79 (2.91, 85.57), p=0.001 [6,13]. This finding is displayed in Figure 7.

Postoperative creatinine abnormality

**Figure 8:**
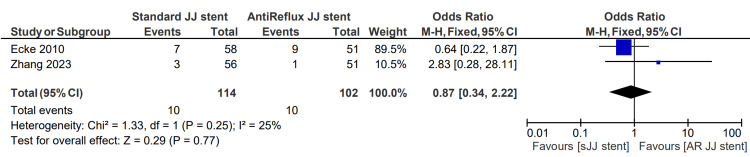
Postoperative creatinine abnormality [6,13] sJJ: standard JJ; AR JJ: antireflux JJ

Postoperative creatinine abnormality was described in two studies, totaling 114 patients in the sJJ stent group and 102 in the AR JJ stent group. The postoperative creatinine abnormality rate for patients having sJJ stents is 8.8%, compared with 9.8% for those with AR JJ stents. The associated OR for postoperative creatinine abnormality was 0.87 (0.34, 2.22) p=0.77 [6,13]. This finding is displayed in Figure 8.

Postoperative hydronephrosis

**Figure 9:**
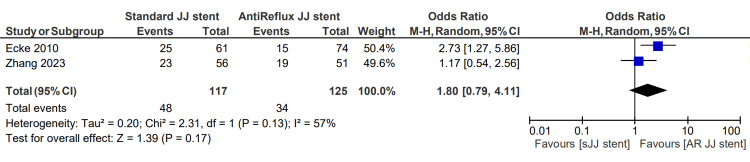
Postoperative hydronephrosis [6,13] sJJ: standard JJ; AR JJ: antireflux JJ

Postoperative hydronephrosis was described in two studies, totaling 117 patients in the sJJ stent group and 125 in the AR JJ stent group. The postoperative hydronephrosis rate for patients with sJJ stents is 41.0%, compared with 27.2% for patients with AR JJ stents. However, this was not statistically significant, with an associated OR of 1.80 (0.79, 4.11), p=0.13 [6,13]. This finding is displayed in Figure 9.

High-grade postoperative hydronephrosis

**Figure 10:**
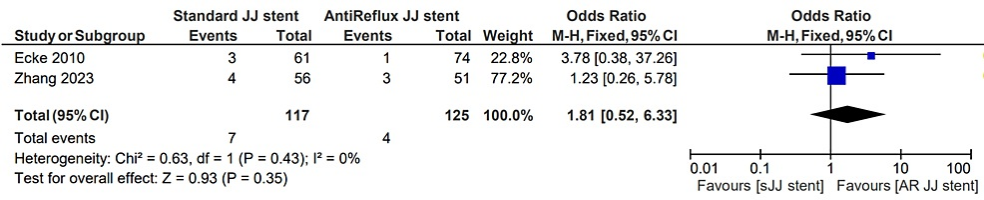
High-grade postoperative hydronephrosis [6,13] sJJ: standard JJ; AR JJ: antireflux JJ

High-grade postoperative hydronephrosis is classified as grade 3 or higher for this meta-analysis. The severity of postoperative hydronephrosis was described in two studies, totaling 117 patients in the sJJ stent group and 125 in the AR JJ stent group. The high-grade postoperative hydronephrosis rate for patients having sJJ stents is 6.0%, compared with 3.2% for patients with AR JJ stents. However, this was not statistically significant, with an associated OR of 1.81 (0.52, 6.33) p=0.35. This finding is displayed in Figure 10 [6,13].

Discussion

In this systematic review and meta-analysis of RCTs, we find that patients undergoing AR JJ stent insertions have a significantly low risk of stent-related symptoms such as stent-related pain and reflux symptoms. These findings are important as they might signify lower emergency departmental visits, medications for pain relief, and a lower rate of stent exchange or removal in patients with AR JJ stents as compared to the sJJ stent.

The pathophysiology of stent-related pain is unknown and likely multifactorial. Hypotheses explaining stent-related pain include distal stent end-causing mucosal irritation, smooth muscle spasms, inflammation, and VUR [2]. The reduction of stent-related symptoms, including stent-related pain and flank pain on urination (reflux symptoms), in our study, mirrors the findings of the RCTs of Ecke et al. and Zhang et al. [6,13]. These findings can be explained by the decrease in the VUR as demonstrated by a cystogram intraoperatively while inserting the JJ stent, with standard stents having reflux 58% of the time and AR JJ stents having reflux 17% of the time in Ritter et al.. These results should encourage the routine use of AR JJ stents. Another strategy to reduce VUR is the complete intraureteral placement of a JJ stent. In a meta-analysis, it was also shown to be effective in reducing SRS in patients undergoing short-term stenting after uncomplicated ureterorenoscopy [13-15]. 

The probable benefits to morbidity, such as decreased analgesics, emergency departmental visits, and JJ stent changes, were not studied in this review as there was a lack of homogenous data. However, inferences can be made from the above results. Moreover, Zhang et al. demonstrated reduced analgesic intake after hospitalization among patients with AR JJ stents, with 25% of the sJJ stent group and 9% of the AR JJ stent group taking analgesics on discharge. Zhang et al. also mentioned that two patients in the sJJ group had presented due to severe pain, and two other patients underwent another operation as the distal end of the sJJ stent had retracted into the ureter. Ecke et al. mentioned that seven patients from the sJJ stent group eventually needed a JJ stent exchange to the AR JJ stent due to SRS. A comparison of quality of life was done by Zhang et al. via EQ-5D-5L scoring, demonstrating significantly higher overall health status index scores among the AR JJ stent group as compared to the conventional JJ stent group. This was due to significantly better "usual activity" scores and "pain/discomfort" scores [13]. The above findings might represent decreased healthcare costs.

We would recommend further RCTs include variables such as analgesics in the community, emergency departmental visits, and further operations due to pain or displacement of ureteric stents to ascertain the morbidity benefit of antireflux JJ stents [6,13].

Limitations

The limitations of this review are that, although this meta-analysis only included RCTs, it is unclear how these patients were randomized, which might lead to selection bias. The study only included English articles, and we might be missing RCTs in other languages. Ritter et al. had a low response rate, which might lead to attrition bias; however, the data from Ritter et al. were only included in the reflux symptoms analysis. There might be a publication bias wherein only positive studies of AR JJ stents would be published [6,13,14]. Although we performed a meta-analysis of short-term SRS, other side effects of urethral stent placements were not included, such as hematuria and urinary tract infections, as they were only included in one of the RCTs. Moreover, long-term side effects of antireflux stents, such as stent encrustations, stone formation, and stent fractures, are not known currently due to the limited utilization of the antireflux stent and subsequent non-existent literature on the topic.

## Conclusions

In conclusion, this systematic review and meta-analysis found that AR JJ stents were associated with a significantly lower risk of stent-related symptoms as compared to sJJ stents, while also not increasing the likelihood of postoperative hydronephrosis. The findings of this study suggest that antireflux ureteral stents may be a preferred option for patients who require ureteral stent placement, especially in patients with ureteral calculi.

It is important to note that this study was limited by the scarcity of studies. More research is needed to confirm the findings of this study and to determine the long-term benefits and risks of antireflux ureteral stents.
